# Healthy ageing: moving forward

**DOI:** 10.2471/BLT.17.203745

**Published:** 2017-11-01

**Authors:** John R Beard, Islene Araujo de Carvalho, Yuka Sumi, Alana Officer, Jotheeswaran Amuthavalli Thiyagarajan

**Affiliations:** a Department of Ageing and Life Course, World Health Organization, avenue Appia 20, 1211 Geneva 27, Switzerland.

Of the many current global challenges, one trend is certain: populations are rapidly ageing and this demographic transition will affect almost all aspects of society. Yet, while we are living longer, it is still unclear whether we live these additional years in good health.^1^^,^^2^ Indeed, older age is characterized by greater diversity in health and circumstances. Even in low-income countries, an 80-year-old person may still be robust and healthy, while a 60-year-old may need significant care and support.^3^ Chronological age is therefore a poor indicator of health status. This diversity in health status is often a consequence of the cumulative impacts of advantage or disadvantage on people’s lives. This means that those with the greatest health needs in older age may have the least access to the resources they require. It also suggests that longer, healthier lives may only be enjoyed by a privileged few.

Yet, when the world united around the *2030 agenda for sustainable development*, governments pledged that no one would be left behind and that everyone would have the opportunity to fulfil their potential in dignity and equality. That this includes older people is explicit in sustainable development goal (SDG) 3, that is to ensure healthy lives and promote wellbeing for all at all ages.^4^

The World Health Organization’s (WHO) *Global strategy and action plan on ageing and health*^5^ provides a policy framework to ensure that the 2030 agenda is inclusive of older people. The strategy builds upon WHO’s new approach to healthy ageing as outlined in the *World report on ageing and health*.^6^ Rather than focusing on the absence of disease, the approach considers healthy ageing from the perspective of the functional ability that enables older people to be, and to do, what they have reason to value. This ability is not only determined by an individual older person’s capacities, but also by the physical and social environments they inhabit.

While the actions outlined in the strategy are likely to be sound investments, to date global funding on healthy ageing has been limited. Patterns of development assistance need to change if we are to ensure everyone has the opportunity to enjoy a long and healthy life. Recent analysis suggests that, relative to disease burden, global funding for health is skewed to younger age groups.^7^ As populations age and the burden of disease shifts increasingly to chronic disorders, this imbalance will become increasingly obvious.^7^^,^^8^

But investment in the health of older people does not need to come at the cost of disinvestment in the health of younger age groups. Older people need health systems that provide integrated and person-centred services, that are located as near as possible to their homes and that deliver care that helps maintain intrinsic capacity for as long as possible. These services are appropriate for all ages and should be the cornerstone of universal health coverage.

In many countries, families are expected to provide the long-term care required by an older person who has significant loss of capacity and needs the help of others to perform basic tasks. This role is most often filled by women – without support or guidance – who can lose professional, financial and educational opportunities as result. 

Creating systems that support caregivers – for example through training, home care or respite care – enables care-dependent older people to live lives of meaning and dignity, while allowing caregivers to pursue other aspirations. It can create jobs and a care economy, and does not necessarily require extensive government funding.

Since physical and social environments are important determinants of health and well-being, another key investment is to ensure that everyone has the opportunity to grow old in age-friendly environments. These environments foster healthy ageing by helping to build and maintain capacity, or by compensating for losses of capacity, for example through flexible transport options. This concept has inspired municipalities in many parts of the world and is reflected in the support for the WHO Global Network of Age-friendly Cities and Communities, which now includes over 500 members in 37 countries.^9^ These communities have benefits for all generations.

Perhaps the most important barriers to ensuring that the SDG’s are inclusive of older people are pervasive misconceptions, negative attitudes and assumptions about older age. Ageism remains socially acceptable, strongly institutionalized, largely undetected and unchallenged. Ageism is a powerful barrier to developing good public policy, because it limits the way issues are framed and addressed. 

Changing how we think, feel and act on age and ageing is therefore a priority. Experience with sexism and racism has shown that changing social norms is possible and can result in more prosperous and equitable societies. Yet, this change requires new ways of thinking. This special issue of the *Bulletin* is one step in achieving this change.
